# Association of blood urea nitrogen with 28-day mortality in critically ill patients: A multi-center retrospective study based on the eICU collaborative research database

**DOI:** 10.1371/journal.pone.0317315

**Published:** 2025-01-14

**Authors:** Ting Deng, Die Wu, Shan-shan Liu, Xing-lin Chen, Zhen-wei Zhao, Lan-lang Zhang

**Affiliations:** 1 Department of Urology, Fuyong People’s Hospital of Baoan District, Shenzhen, Guangdong Province, China; 2 Department of Chinese Medicine and Anorectology, Fuyong People’s Hospital of Baoan District, Shenzhen, Guangdong Province, China; 3 Department of Nursing, Fuyong People’s Hospital of Baoan District, Shenzhen, Guangdong Province, China; 4 Department of Epidemiology and Biostatistics, Empower U.X&Y Solutions Inc, Boston, Massachusetts, United States of America; 5 Department of Haemodialysis, Fuyong People’s Hospital of Baoan District, Shenzhen, Guangdong Province, China; Ataturk University Faculty of Medicine, TÜRKIYE

## Abstract

**Objective:**

Blood urea nitrogen (BUN) is a commonly used biomarker for assessing kidney function and neuroendocrine activity. Previous studies have indicated that elevated BUN levels are associated with increased mortality in various critically ill patient populations. The focus of this study was to investigate the relationship between BUN and 28-day mortality in intensive care patients.

**Methods:**

This was a multi-centre retrospective cohort study that made use of data from the eICU Collaborative Research Database. The primary exposure variable was BUN, and the outcome was 28-day mortality. The following variables were included as covariates: age, gender, BMI, white blood cell count, creatinine, GCS score, APACHE IV score, and diabetes. The statistical analyses included univariate and multivariate logistic regression, as well as generalized additive modelling, which was employed to assess the non-linear relationship between BUN and mortality.

**Results:**

A total of 63,757 elderly patients were included in the study, with a 28-day mortality of 6.5%. The univariate analysis indicated that elevated BUN quartiles were associated with an increased risk of mortality. The results of the multivariate analysis further confirmed the non-linear relationship between BUN and mortality. When BUN was less than 32 mg/dL, there was a significant positive association, with an adjusted odds ratio of 1.230 (95% CI: 1.154–1.311, p<0.0001) for every 10 mg/dL increase in BUN. However, when BUN was greater than or equal to 32 mg/dL, BUN level had no significant effect on mortality.

**Conclusion:**

BUN showed a nonlinear, threshold correlation with 28-day mortality in critically ill patients. The higher the BUN, the greater the risk of death if the BUN is below the threshold.

## Introduction

Blood urea nitrogen (BUN) is a waste product of protein metabolism produced by the liver and excreted by the kidneys [1]. As a biomarker, BUN is commonly used for the routine assessment of kidney function [2]. However, BUN is not solely a marker of renal function. In addition to assessing renal function, BUN is also a useful indicator of neurohormonal activity [3]. This is because impaired cardiac and renal function, as well as neurohormonal dysregulation, can lead to increased BUN levels, which have been associated with higher mortality across a variety of diseases [3, 4].

A number of studies have demonstrated a significant association between elevated BUN levels and mortality in a variety of critically ill patients, including those with heart failure [5], acute coronary syndrome [6], acute exacerbation of chronic obstructive pulmonary disease (COPD) [7], COVID-19 [8, 9], and acute pancreatitis [10, 11] and ICU patients [12]. A study demonstrated that elevated BUN levels were significantly associated with mortality in critically ill ICU patients, and this association persisted even after adjusting for multiple other potential confounding factors [9]. A positive correlation between BUN levels and in-hospital mortality was also confirmed in a study [12], but the AUC value of 0.63 for BUN derived from ROC analysis indicated that it has limited predictive capacity. Furthermore, there are discrepancies in the BUN threshold levels identified by different studies [7, 9], which may be attributable to the heterogeneity of the study populations and the statistical methodologies employed.

We hypothesised that both high and low BUN levels are associated with a high risk of all-cause mortality within 28 days in critically ill patients. Therefore, the aim of this study was to investigate the relationship between BUN levels and death and to explore the threshold of BUN levels where the risk of death is significantly increased, which is a high priority for critically ill patients.

## Methods

### Data source

The data analyzed in this study were extracted from the eICU-CRD [13]. The database was a multi-center ICU database for over 200,000 admissions in 2014 and 2015 at 208 United States Hospitals. All data were automatically stored via the Philips Healthcare eICU program and retrieved electronically. The eICU-CRD has been employed for observational research [14–16]. The utilisation of this database has been approved by the institutional review boards of Massachusetts Institute of Technology (Cambridge, MA, USA). One author (Xinglin Chen) obtained the access and was responsible for the data extraction (certification number:40859994). All methods were performed in accordance with the eICU-CRD relevant guidelines and regulations. As the study was retrospective and analysed anonymous data, informed consent was not required.

### Study population

**Fig 1:**
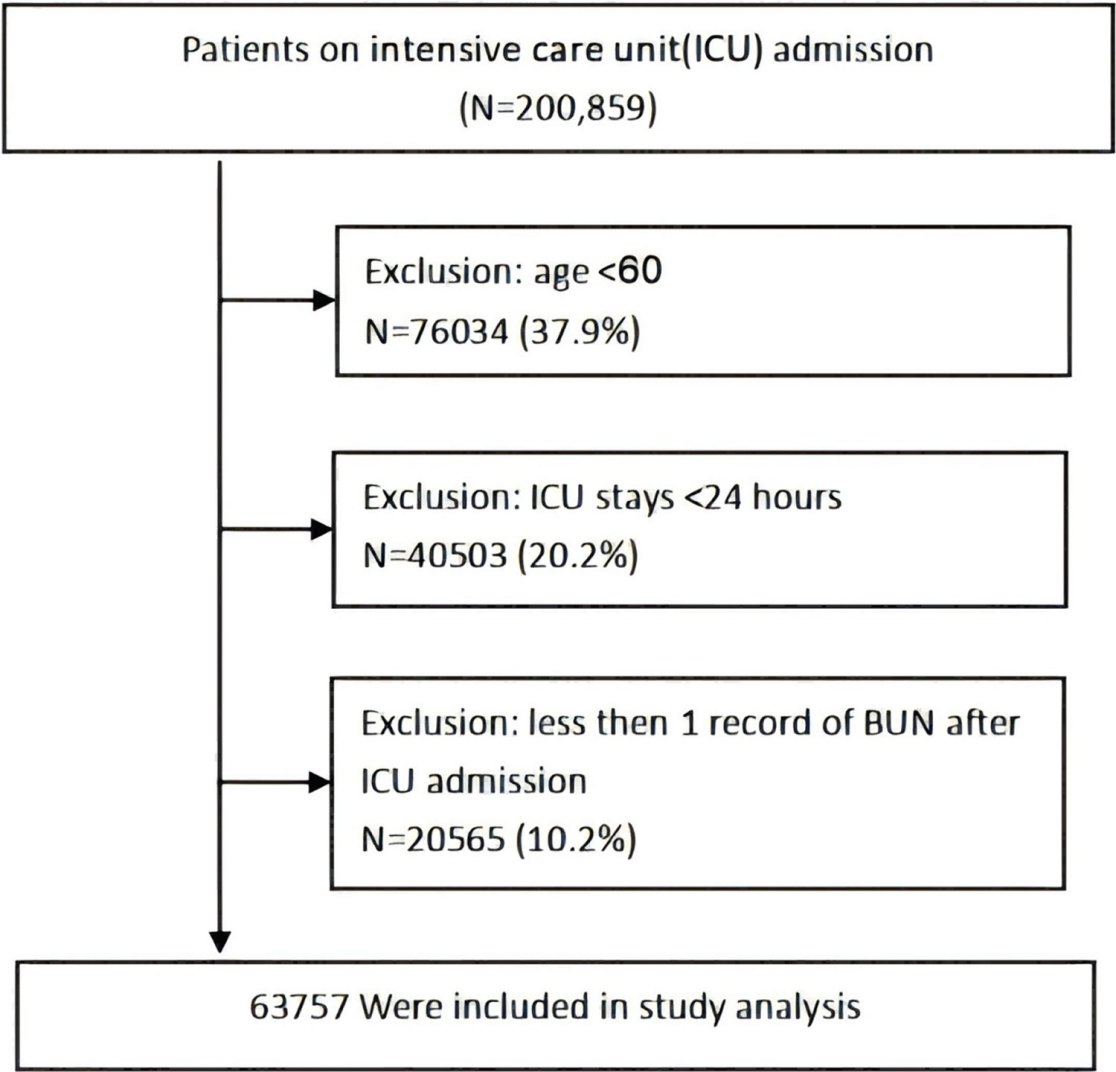
Flow chart of study population. ICU, intensive care unit.

This study was a multi-center retrospective cohort study. The study initially involved 200859 participants; 137102 participants were subsequently excluded from this study, leaving 63757 cases for the final data analysis. The exclusion criteria were as follows (1) less than 60 years of age; (2) admission to ICU for less than 24 hours; (3) less than one record of BUN after ICU admission. The study flow chart was shown in (Fig 1).

### Variables

The eICU database comprises a range of data, including demographic information, physiological indicators derived from bedside monitors, diagnoses according to the International Classification of Diseases, 9th Edition, Clinical Modification (ICD-9-CM) codes, and other laboratory data obtained during routine medical care. All data were collected within 24 hours of admission.

The primary exposure variable was BUN, which was quantified in mg/dL. The covariates included in this study were selected on the basis of clinical experience and risk factors reported in the literature. The involved covariates were age (age at admission), gender (male or female) and body mass index (BMI, kg/m^2^). Additionally, white blood cell (WBC) counted in units per microlitre of blood was considered an indicator of inflammation and infection, while serum creatinine level in mg/dL was applied for renal function assessment. The Glasgow Coma Scale (GCS) was employed to evaluate the level of consciousness, with scores ranging from 3 to 15, with lower scores indicating a more severe impairment of consciousness. The Acute Physiological and Chronic Health Evaluation (APACHE) IV score was utilized to assess the severity of the condition, with higher scores indicating a more severe condition. The status of diabetes mellitus (DM) was categorised as present or absent based on the patient’s medical history.

### Outcomes

The study’s end point was 28-day mortality, which was defined as death occurring within 28 days of ICU admission.

### Statistical analysis

Descriptive statistics were utilized to analyse the characteristic and distribution of the population. Continuous variables are presented as mean standard deviation (Gaussian distribution) or median and interquartile range (IQR) (skewed distribution), and categorical data are described as numbers and percentages. Differences among the tertiles of BUN were assessed using one-way analysis of variance (ANOVA) for continuous data with a Gaussian distribution, the Kruskal-Wallis H test for skewed continuous data, and chi-squared tests for categorical variables.

A generalized additive model (GAM) was employed to ascertain the dose-response relationship between BUN and 28-day mortality, and smoothed curve-fitting plots were generated, as described in the previous analysis [17, 18]. Univariate and multivariate binary logistic regression models were used to test the connection between the BUN and 28-day mortality. Furthermore, two adjustment models were constructed to account for potential confounding variables. These included age (years), gender, BMI, WBC, creatinine, Apache IV score, GCS score, and diabetes. The results are presented as odds ratios (ORs) with its 95% confidence intervals (95% CIs).

Subsequently, a two-piece-wise linear regression model was employed to assess the threshold effect of BUN on 28-day mortality. The turning point for BUN was identified using “exploratory” analyses, which entailed moving the trial turning point along the pre-defined interval and selecting the one that yielded the maximum model likelihood. Additionally, a log-likelihood ratio test was performed, comparing the one-line linear regression model with the two-piecewise linear model. All the statistical analysis was performed using R software version 4.0.0 (http://www.r-project.org) and the Empower Stats (www.empowerstats.com, X&Y solutions, Inc. Boston MA). Two-sided p values < 0.05 was considered statistically significant.

### Ethics approval and consent to participate

Data was extracted from the eICU Collaborative Research Database (eICU-CRD)9 in accordance with the data usage agreement (our record ID: 40859994) by the PhysioNet review committee. The utilized database is released under the Health Insurance Portability and Accountability Act (HIPAA) safe harbor provision. This was a retrospective analysis based on an anonymous database for researchers and did not require ethical approval from the local ethics committee.

## Results

### Baseline characteristics

Data from 63,757 patients were analyzed (Table 1). All participants were adults aged 60–89 years old (mean±SD: 73.87±8.67 years),34170 patients were females (53.6%). The 28-day ICU mortality rate was 4175/63757 = 6.5% (95% CI; 6.36–6.74). In comparison to participants in the lowest tertile of BUN, those in the highest tertile were older, higher BMI, and exhibited a significantly elevated prevalence of DM. Additionally, the male proportion significantly decreased from 51.25% to 43.29% (P < 0.001), indicating a significant difference in gender distribution.

**Table 1:** Baseline characteristics of participants (n = 63757).

Characteristics	BUN (per 10 mg/dL)			
	Tertile 1	Tertile 2	Tertile 3	*P*-value
	0.10–1.79	1.80–3.19	3.20–25.40	
	n = 20081	n = 21928	n = 21748	
Age (years)	71.90 ±8.30	74.53±8.64	75.02 ±8.74	<0.001
Gender, n (%)				<0.001
Male	10289 (51.25)	9873 (45.04)	9415 (43.29)	
Female	9789 (48.75)	12049 (54.96)	12332 (56.71)	
BMI (kg/m^2^)	27.90 ±7.08	28.61 ±7.55	29.34 ±8.51	<0.001
APACHE IV score	54.29 ±19.16	65.89 ±21.63	78.98 ±23.50	<0.001
GCS Score	12.92±3.41	12.73±3.52	12.46 ±3.57	<0.001
WBC (10^9/L)	6.50 (5.20–7.52)	10.59 (9.46–12.10)	18.20(15.60–22.60)	<0.001
Creatinine (mg/dL)	0.70 (0.59–0.79)	1.12 (0.99–1.29)	2.30 (1.78–3.50)	<0.001
DM, n (%)				<0.001
No	18222 (90.74)	19262 (87.84)	18050 (83.00)	
Yes	1859 (9.26)	2666 (12.16)	3698 (17.00)	

Results in table: Mean± SD, Median (Q1−Q3)/ n (%).

Abbreviations: *BUN*, blood urea nitrogen; *BMI*, body mass index; *APACHE*, Acute Physiological and Chronic Health Evaluation; *GCS*, Glasgow Coma Scale; *WBC*, white blood cell; DM, diabetes mellitus. Among the 63757 participants, the amount of missing values for the covariates were 10 (0.02%) for gender, 1935 (3.03%) for BMI, 6405 (10.05%) for APACHE IV score, 1036 (1.62%) for GCS Score, 4342 (6.81%) for WBC, 108 (0.17%) for Creatinine.

### Univariate analysis for 28-day mortality

Table 2 shows the univariate logistic models. The analysis revealed that those in the highest BUN tertile (3.20–25.40 mg/dL) exhibited a significantly elevated risk of mortality in comparison to subjects in the lowest BUN tertile (0.10–1.79 mg/dL). The OR for the middle tertile (1.80–3.19 mg/dL) was 1.99 (95% CI: 1.80–2.20, P < 0.0001), while the OR for the highest tertile (3.20–25.40 mg/dL) was 3.85 (95% CI: 3.52–4.23, P < 0.0001). Covariates such as APACHE IV score, WBC, and creatinine levels were significantly positively correlated with the 28-day mortality, whereas the GCS score showed a significant negative correlation.

**Table 2:** Univariate analysis for 28-day mortality.

Exposure	Statistics	OR (95%CI)	*P-*value
BUN (per 10 mg/dL)	3.09 ± 2.30	1.16 (1.15, 1.17)	<0.0001
BUN Tertile (per 10 mg/dL)			
Tertile 1 (0.10–1.79)	20081 (31.50)	Reference	
Tertile 2 (1.80–3.19)	21928 (34.39)	1.99 (1.80, 2.20)	<0.0001
Tertile 3 (3.20–25.40)	21748 (34.11)	3.85 (3.52, 4.23)	<0.0001
Age (years)	73.87 ± 8.67	1.01 (1.01, 1.02)	<0.0001
Gender, n (%)			
Male	29577 (46.40)	Reference	
Female	34170 (53.60)	1.03 (0.96, 1.09)	0.4008
BMI (kg/m^2^)	28.64 ± 7.77	0.99 (0.99, 1.00)	0.0049
APACHE IV score	66.71 ± 23.78	1.04 (1.04, 1.04)	<0.0001
GCS score	12.70 ± 3.51	0.83 (0.83, 0.84)	<0.0001
WBC(10^9/L)	12.61 ± 8.51	1.03 (1.03, 1.04)	<0.0001
Creatinine(mg/dL)	1.62 ± 1.57	1.15 (1.14, 1.17)	<0.0001
DM, n (%)			
No	55534 (87.10)	Reference	
Yes	8223 (12.90)	1.03 (0.94, 1.13)	0.518

Results in table: Mean± SD / n (%).

Abbreviations:*BUN*, blood urea nitrogen; *BMI*, body mass index; *APACHE*, Acute Physiological and Chronic Health Evaluation; *GCS*, Glasgow Coma Scale; *WBC*, white blood cell; *DM*, *diabetes mellitus; OR*, odds ratio; *CI*, confidence interval.

### Relationship between BUN and 28-day mortality

In the unadjusted model, for each 10 mg/dL increase in BUN, the OR for mortality was 1.157 (95%CI 1.146, 1.169, P<0.00001). This finding remained significant even in the adjusted models (OR = 1.029, 95%CI 1.010, 1.049, P = 0.00262), indicating that higher BUN is independently associated with a higher risk of mortality. Analysis by BUN tertiles further presented those patients in the middle and highest tertiles of BUN had a significantly higher mortality risk compared to those in the lowest tertile. Specifically, the OR for mortality were 1.181 (95%CI 1.050, 1.327, P = 0.00545) for the middle tertile and 1.413 (95%CI 1.245, 1.603 P<0.00001) for the highest tertile (Table 3).

**Table 3:** Relationship between BUN and 28-day mortality.

Outcome	Non-adjusted model (OR, 95%, *P*)	Adjusted model I (OR, 95%, *P*)	Adjusted model II (OR, 95%, *P*)
BUN (per 10 mg/dL)	1.157 (1.146, 1.169) <0.00001	1.156 (1.144, 1.168) <0.00001	1.029 (1.010, 1.049) 0.00262
BUN tertile			
Low	Reference	Reference	Reference
Middle	1.991 (1.803, 2.198) <0.00001	1.962 (1.776, 2.167) <0.00001	1.181 (1.050, 1.327) 0.00545
High	3.855 (3.516, 4.226) <0.00001	3.795 (3.459, 4.165) <0.00001	1.413 (1.245, 1.603) <0.00001

Abbreviations: *OR*, odds ratio, *BUN*, blood urea nitrogen.

ModelⅠadjusted for Gender and Age(years).

ModelⅡadjusted for age (years), gender, BMI, WBC, creatinine, Apache IV score, GCS score

and diabetes.

### Identification of nonlinear relationship

**Fig 2:**
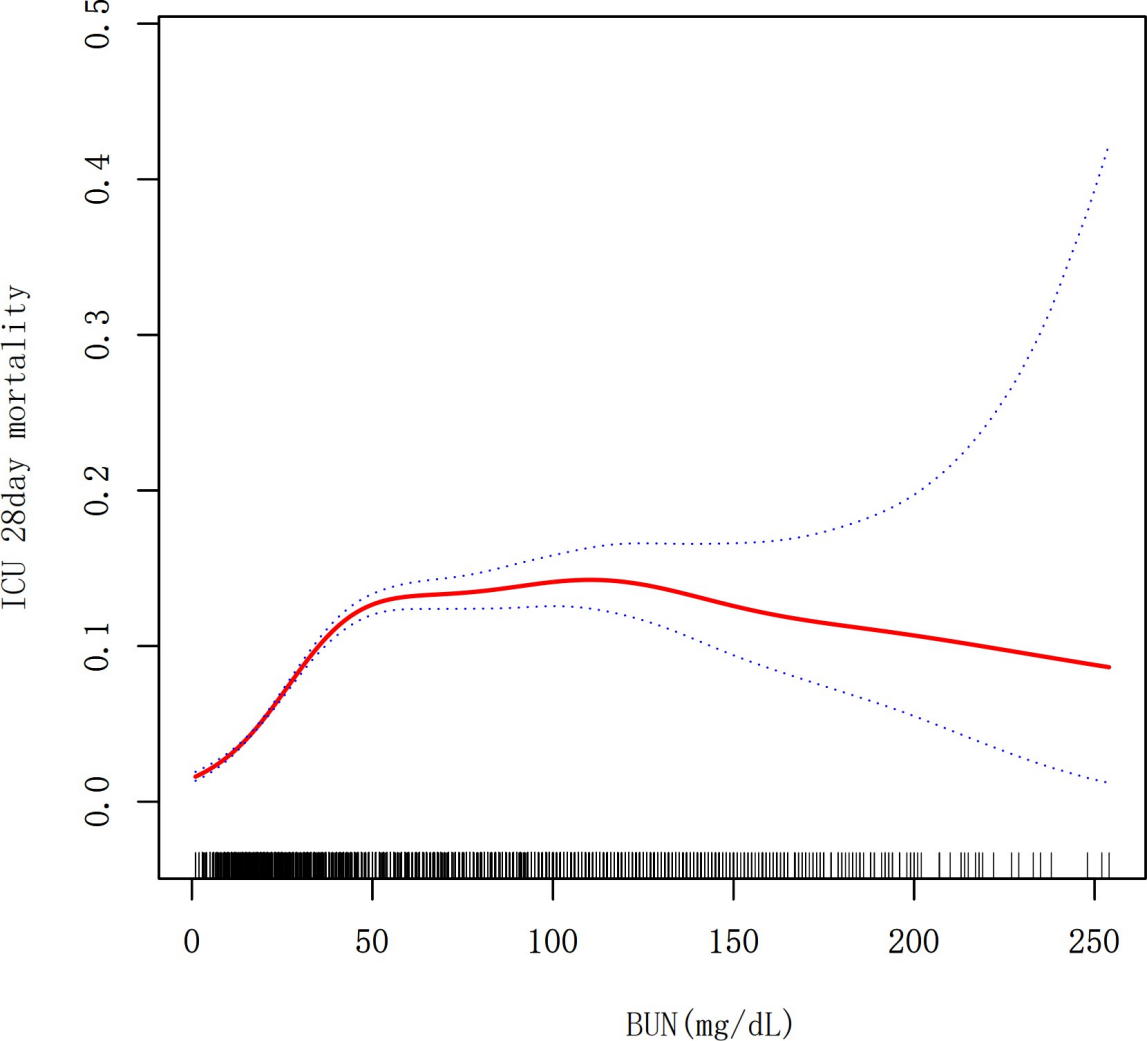
Associations between the BUN(mg/dL) and 28-day mortality in critically ill patients. A threshold, nonlinear association between the BUN and 28-day mortality was found in a generalized additive model (GAM). Solid rad line represents the smooth curve fit between variables. Blue bands represent the 95% of confidence interval from the fit. Adjusted for age (years), gender, BMI, WBC level, creatinine level, GCS score, APACHE IV score and DM.

A nonlinear dose–response relationship was presented between the BUN and mortality (Fig 2 and Table 4). When the BUN was <32mg/dL, a positive correlation was observed between the BUN and mortality, with an adjusted OR of 1.230 (95% CI: 1.154–1.311, *P* < 0.0001) for each 10 mg/dL in the BUN. When the BUN was ≥32mg/dL, the effect was not significant, as indicated by an adjusted OR of 0.997 (95% CI: 0.975–1.020, *P* = 0.811), suggesting negligible impact on mortality (Table 4). Utilizing the generalized additive model, a nonlinear association between the BUN and 28-day mortality was identified (Table 4). A comparison was conducted between the linear regression model and a two-piece-wise linear regression model, and the *P* value of the log-likelihood ratio test was less than 0.001. This result demonstrates that the two-piece-wise linear regression model should be used to fit the model.

**Table 4:** Threshold effect analysis of the BUN(mg/dL)and 28-day mortality.

Models	OR (95%CI)	*P* value
Model I		
One line effect	1.029 (1.010, 1.049)	0.0026
Model II		
Turning point (K)	3.2 mg/dL	
BUN(per 10 mg/dL) < K	1.230 (1.154, 1.311)	<0.0001
BUN(per 10 mg/dL) ≥ K	0.997 (0.975, 1.020)	0.811
*P* value for LRT test*		<0.001

Data were presented as OR (95% CI) *P* value; Model I, linear analysis; Model II, non-linear analysis. Adjusted for age (years), gender, BMI, APACHE IV score, GCS Score, WBC, Creatinine, DM. Abbreviations: *OR*, odds ratio; *CI*, confidence interval; *BUN*, blood urea nitrogen; *LRT*, logarithm likelihood ratio test. * *P*<0.05 indicates that model II is significantly different from Model I.

## Discussion

The objective of this study was to investigate the relationship between BUN levels and 28-day mortality in critically ill patients. The results demonstrated a nonlinear correlation between BUN and 28-day mortality in critically ill patients, with the formation of a threshold effect curve. Additionally, distinct correlations between BUN and 28-day mortality were observed on either side of the inflection point. When BUN was below 32 mg/dL, the risk of death was significantly increased by 23% for every 10 mg/dL increase in BUN (adjusted OR = 1.230, 95% CI: 1.154–1.311, P<0.0001). This suggests that healthcare professionals should pay close attention to patients with low BUN, as low BUN levels may indicate some potentially serious conditions. Conversely, elevated BUN had no significant effect on mortality when BUN was greater than 32 mg/dL (adjusted OR = 0.997, 95% CI: 0.975–1.020, P = 0.811). When the BUN levels were categorized into three groups, the risk of death in the moderate and high BUN groups was 1.962 and 3.795 times higher, respectively, than that in the low BUN group. These differences were all statistically significant. These findings indicate that BUN may serve as a valuable biomarker for prognostication in critically ill patients, enabling clinicians to identify high-risk individuals and implement targeted therapeutic strategies in a timely manner.

Numerous studies have confirmed that elevated BUN levels are associated with a poor prognosis for a variety of serious diseases. Mohan et al. [19] observed that higher BUN levels were associated with a significantly increased risk of in-hospital mortality in critically ill AECOPD patients. Bernhard et al. [12] also found that BUN was significantly associated with in-hospital mortality (HR 1.03; 95% CI 1.01–1.05; p < 0.001), and this association persisted even after correction for several factors such as APACHE2 and renal function. Canlin et al. [6] observed an association between elevated BUN concentrations and an increased risk of cardiovascular disease and all-cause mortality. Yaser et al. [20] and colleagues also identified baseline BUN levels as an independent predictor of short-term mortality in patients with acute pulmonary embolism. Hongfang et al. [21] further demonstrated a nonlinear association between BUN and all-cause mortality and cardiovascular disease mortality in diabetic patients. A systematic evaluation [22] demonstrated that elevated BUN levels were an independent predictor of all-cause mortality in patients with heart failure. Additionally, a study by Jeffrey M. et al. [23] indicated that patients with BUN levels above the median exhibited a significantly increased mortality rate when treated with high-dose labelled diuretics. The studies by Jun Liu [24] and Jiarui Zhang [7] also lend support to the view that BUN plays an independent role in determining the severity of acute disease and the likelihood of poor prognosis, particularly in patients with acute aortic coarctation and AECOPD, where elevated BUN levels have been shown to be significantly associated with in-hospital mortality. Our research builds upon previous research in the same field, but with greater precision. In addition to identifying a non-linear relationship, we have also determined the threshold value.

In addition, a study [25] included 4176 patients (67±13 years) admitted to ICUs in Germany between 2004 and 2009, and patients were retrospectively followed up from May 2013 to November 2013. Cox regression was used to analyse the relationship between hospital BUN and in-hospital and long-term mortality. The study revealed that elevated BUN levels at the time of admission were significantly linked to unfavourable outcomes in critically ill ICU patients. Furthermore, the researchers identified an optimal threshold of 28 mg/dL, above which patients exhibited a poor prognosis. Our study aligns with this one in terms of its overall objective, but we employed a generalised additive model to assess the nonlinear relationship between BUN and 28-day mortality. Furthermore, we evaluated the predictive effects of linear and segmented linear models, with our thresholds being more precise. Furthermore, our study provides the first insight into the dose-response relationship between BUN levels and short-term prognosis in a large population of ICU patients.This provides a crucial foundation for clinicians to assess the prognosis of critically ill patients and make treatment decisions.

Elevated BUN levels have been linked to an increased risk of mortality through a number of mechanisms. Primarily, BUN serves as an important indicator of renal function. When renal function is impaired, the kidneys are unable to effectively remove metabolic waste products, leading to elevated BUN levels. Prolonged renal insufficiency has been shown to trigger systemic metabolic disorders [25] and increase the risk of cardiovascular events. Secondly, in patients with heart failure, elevated BUN levels may be indicative of a decline in the heart’s pumping function, which can result in impaired blood return, thereby increasing the burden on the heart and potentially exacerbating heart failure [26]. Furthermore, elevated BUN levels may indicate increased protein catabolism or decreased renal function, which can result in metabolic disturbances such as electrolyte imbalance and acid-base balance imbalance. These in turn can lead to further impairment of organ function [27]. It has been demonstrated that elevated BUN levels are associated with an inflammatory response within the body. This inflammatory response may subsequently lead to vascular endothelial dysfunction and thrombosis, thereby increasing the risk of cardiovascular events [28]. Accordingly, elevated BUN levels may elevate the risk of mortality through a number of pathways that affect renal function, cardiac function, and systemic metabolic status. It is imperative that patients exhibiting markedly elevated BUN levels be subjected to rigorous monitoring of renal function and cardiac status. In addition, the implementation of efficacious therapeutic measures is of paramount importance to enhance the prognosis.

The present study is characterized by a number of key strengths. Firstly, the reliability of the data is attributable to the relatively large sample size, as this was a multi-centre study encompassing patient data from 208 hospitals in the United States. Secondly, a nonlinear relationship was observed between BUN and the 28-day risk of death. Furthermore, we identified the inflection point at which BUN was positively correlated with mortality when BUN was less than 32 mg/dL. This provides a crucial basis for clinicians to assess the prognosis of critically ill patients and make treatment.

### Study limitations

This study also has some potential limitations. Firstly, this was a retrospective analytical study, and although we have adjusted for some confounders, there may be residual or unmeasured confounders. Secondly, it should be noted that all patients included in this study were from the intensive care unit. As a result, the findings may not be applicable to patients in general wards. Also because we included a population aged 60–89 years. Therefore, our results cannot be extrapolated to other age groups. Furthermore, the study is subject to additional limitations, including the absence of data for certain variables. However, the use of modern methods to deal with missing data has been employed in order to minimize the potential for bias. Furthermore, it was not possible to ascertain long-term outcomes, as the database only provided data on short-term follow-up. In conclusion, the study data were derived from a single database, which may limit the generalizability of the results. It is therefore recommended that future prospective studies be conducted in different populations in order to further validate and extend the applicability of these findings.

## Conclusions

BUN showed a nonlinear, threshold correlation with 28-day mortality in critically ill patients. The higher the BUN, the greater the risk of death if the BUN is below the threshold.
